# Network analysis of mental health knowledge and stigma among high school students in Sichuan, China

**DOI:** 10.3389/fpsyt.2026.1728556

**Published:** 2026-01-29

**Authors:** Lie Zhou, Xiao-Peng Deng, Cong Wang, Yi-Yue Yang, Yi-Hao Liu, Hui Jin, Yang Wen, Yun Xiao, Jawad Ahmad, Jia Cai, Bo Liu, Xin-Feng Zhang, Zong-Ling He, Xiao-Fei Zhou, Xin-Ling Wang, Zhe-Ming Tu, Mao-Sheng Ran

**Affiliations:** 1 National Center for Mental Disorder, West China Hospital, Sichuan University, Chengdu, Sichuan, China; 2 Department of Social Psychiatry, West China Hospital, Sichuan University, Chengdu, Sichuan, China; 3 Mental Health Center of Yangtze University, Jingzhou, Hubei, China; 4 Jingzhou Mental Health Center, Jingzhou, Hubei, China; 5 Chengdu Fourth People’s Hospital, Chengdu, Sichuan, China; 6 Jingzhou Experimental Middle School, Jingzhou, Hubei, China

**Keywords:** China, mental health knowledge, network analysis, stigma, youth students

## Abstract

**Background:**

Lack of mental health knowledge (MHK) and stigma toward mental illness pose significant barriers to help-seeking behaviors among Chinese high school students, amid intense academic pressures and cultural influences. Traditional aggregate scoring methods overlook dynamic interconnections between specific knowledge items and stigma attitudes. This study applies network analysis to model MHK and stigma as interconnected systems, identifying central nodes, bridges, and potential intervention targets in a large adolescent sample.

**Methods:**

A cross-sectional survey was conducted among 12,537 high school students in Sichuan Province, China, between October 2024 and January 2025.The MHK was assessed using the 20-item Mental Health Knowledge Questionnaire (MHKQ), and the stigma via the 12-item Perceived Devaluation and Discrimination Scale (PDD). Networks were estimated with the IsingFit algorithm in R (v4.3.2), incorporating partial correlations. Centrality (strength), bridge expected influence, and stability were computed. The NodeIdentifyR algorithm (NIRA) was used to simulated aggravating and alleviating interventions on network sum scores. Gender invariance was tested using the NetworkComparisonTest package.

**Results:**

The network revealed two communities (MHK and stigma) with dense intra-cluster connections and key bridges. MHKQ11 (“optimistic attitude, good relationships, and healthy habits help maintain mental health”) showed the highest centrality (strength: 12.06), serving as a MHK hub. MHKQ10 (“short-term medication suffices for severe mental illnesses like schizophrenia without long-term adherence”) bridged to stigma items (e.g., Stigma5: "most employers will not hire a person who has been hospitalized for mental illness"; bridge expected influence: 0.957). Network stability was robust (CS > 0.672). Aggravating simulations were associated with the highest sum scores for MHKQ10, MHKQ13, and MHKQ14. Alleviating interventions showed the greatest potential for score reduction via MHKQ11, MHKQ8, and MHKQ16. Gender networks showed invariance (global strength difference: 0.37, p = 0.693).

**Conclusions:**

This network analysis highlights MHKQ10 and MHKQ11 as pivotal targets for stigma reduction, with misconceptions about treatment adherence linking knowledge deficits to devaluation perceptions. Gender-invariant structures suggest universal applicability for school-based interventions, aligning with China’s mental health initiatives to enhance literacy and promote equity.

## 1 Introduction

Mental disorders among adolescents represent a significant public health challenge worldwide, with increasing prevalence rates underscoring the need for early intervention and education. In China, where high school students face intense academic pressures and rapid social changes, mental health issues such as depression, anxiety, and psychotic disorders are particularly pronounced, affecting up to 20-30% of this population (1). Despite growing awareness, low levels of mental health knowledge (MHK) and persistent stigma toward mental illness remain critical barriers to help-seeking behaviors, treatment adherence, and social integration. Stigma, often manifesting as perceived devaluation and discrimination, not only exacerbates the burden on affected individuals but also perpetuates misconceptions within communities, including educational settings (2). For instance, studies have shown that among Chinese adolescents, the awareness rate of mental health knowledge is only around 66% (3), which falls significantly short of national health goals and is associated with heightened stigma levels.

Prior studies using conventional regression and factor analyses have consistently established a negative correlation between overall mental health literacy and stigmatizing attitudes (4, 5). While these aggregate-level findings provide a foundational understanding, traditional approaches often rely on sum scores, treating these constructs as latent variables. However, such methods overlook the dynamic interconnections between specific knowledge items and stigma attitudes, potentially missing nuanced pathways for intervention. Network analysis, an emerging framework in psychiatric research, models psychological phenomena as interconnected systems of nodes and edges, allowing for the identification of central elements, bridges between domains, and simulation of interventions. This approach has been successfully applied to symptom networks in depression and anxiety among adolescents, revealing key hubs that influence overall psychopathology. Yet, its application to MHK and stigma in Chinese youth remains limited, with few studies exploring gender differences or intervention targets. Recent investigations highlight that low mental health literacy in China is linked to stigma and discrimination, potentially hindering help-seeking and relating to poorer outcomes, particularly in high school settings where academic stress compounds these issues (6, 7).

In China, cultural factors such as collectivism and face-saving may amplify stigma, which may correlate with underutilization of mental health services among students. Prior research indicates that misconceptions about the incurability of mental illnesses or the irrelevance of psychological problems to academic performance are prevalent, particularly in rural or high-pressure educational environments. For example, surveys among Chinese high school students have revealed that mental health problems are common, with factors like the COVID-19 pandemic exacerbating academic performance-related stress (8). Gender disparities further complicate this landscape, with females often reporting higher stigma internalization due to societal expectations (9), though empirical network comparisons are scarce. Moreover, stigma has been linked to increased depression rates in this demographic (10), making it challenging for students to seek help and highlighting the need for culturally sensitive interventions. Addressing these gaps is essential for developing targeted school-based programs to enhance MHK and reduce stigma, ultimately promoting mental health equity. Bilingual populations, such as Chinese-English speakers, may exhibit unique stigma patterns influenced by cross-cultural exposures, further emphasizing the diversity within Chinese adolescent groups (6).

The present study using network analysis aimed to examine the interconnections between MHK and perceived stigma among Chinese high school students, identifying central nodes, bridge symptoms, and potential intervention points through threshold perturbations. We hypothesize that core knowledge deficits will bridge to stigma perceptions, with gender-invariant structures suggesting universal applicability of findings. This research may inform evidence-based strategies for stigma reduction in adolescent populations, aligning with national mental health initiatives in China.

## 2 Methods

### 2.1 Study design and participants

A cross-sectional design was used, targeting high school students from 15 schools in Sichuan Province, China. From October 2024 to January 2025, a total of 12,537 valid questionnaires were collected, yielding a response rate of 93.03%. Data quality control procedures were rigorously implemented. Participation was voluntary, confirmed via an initial consent item; students who declined were excluded immediately. To prevent missing data, the online survey platform was configured to require a response for each item before proceeding or submitting. Furthermore, to ensure data validity and filter out inattentive responses, questionnaires were screened based on completion time; specifically, responses with an average duration of less than 2 seconds per (11) item were excluded from the final analysis. The participants were aged 14 to 19 years (M = 16.88, SD = 1.04) and included 4974 students in Grade 10, 5503 in Grade 11, and 2060 in Grade 12 (4,877 males and 7,660 females). Detailed demographic characteristics are summarized in **Table 1**. Teachers in schools were trained on survey instrument administration, ensuring adherence to guidelines and addressing common issues. Trained teachers distributed questionnaires to students and supervised their completion. All participants received detailed online study information and provided informed consent. The study protocol was approved by the Biomedical Research Ethics Committee of West China Hospital, Sichuan University (Approval No.: 2022-1790).

**Table 1:** Demographic characteristics of the study participants (N = 12,537).

Characteristic	N = 12,537^1^
Age	16.88 (1.04)
Sex	
Females	7,660 (61.10%)
Males	4,877 (38.90%)
Ethnicity	
Han	11,579 (92.36%)
Other	958 (7.64%)
Grade	
Grade 10	4,974 (39.67%)
Grade 11	5,503 (43.89%)
Grade 12	2,060 (16.43%)
Domicile	
Rural	2,451 (19.55%)
Urban	10,086 (80.45%)
Accommodation Type	
Boarding School	2,257 (18.00%)
Day School	10,280 (82.00%)

^1^Mean (SD); n (%).

### 2.2 The 20-item Mental Health Knowledge Questionnaire

The MHKQ was used to assess the mental health knowledge levels of adolescents in this survey. This questionnaire was developed by the Chinese Ministry of Health in 2009 and has been widely employed to evaluate mental health knowledge levels among Chinese populations (12), including adolescents. The scale covers multiple content domains, including general concepts of mental health, warning signs of disorders, and developmental issues specific to adolescence, allowing for a multifaceted assessment of mental health literacy. Items 1–16 were answered with “Yes” or “No”, while items 17–20 were answered with “Know” or “Don’t know”. For the data analysis, all items were recoded into a binary format: correct answers were coded as 1, while incorrect responses or “don’t know” were coded as 0. According to previous studies, the Cronbach’s α coefficient of the MHKQ was 0.57 to 0.73 (13). In the present study, the Cronbach’s α coefficient of the MHKQ was 0.853. Although previous studies reported moderate internal consistency (0.57–0.73), network analysis does not rely on the assumption that items are reflective indicators of a single latent variable. Instead, it conceptualizes items as autonomous components within a complex system, making item-level interactions meaningful even if global internal consistency varies.

### 2.3 Perceived Devaluation and Discrimination Scale

The 12-item Perceived Devaluation and Discrimination Scale (PDD) was used to evaluate perceived stigma toward mental illness and patients with mental illness among adolescents in this survey. The PDD was originally developed by Link et al. in 1987 (14), and its Chinese version has been widely used to assess perceived public stigma in Chinese populations. Items were answered on a 4-point Likert scale from “strongly agree” to “strongly disagree.” To facilitate network estimation using the IsingFit algorithm, which is specifically designed for binary data, PDD item responses were dichotomized. According to previous studies, the Cronbach’s α coefficient of the PDD was 0.863 (15, 16). In the current study, the Cronbach’s α coefficient of the PDD was 0.886.

### 2.4 Data analysis

Descriptive statistical analyses and network analysis modeling were performed in R Studio (version 4.3.2) using R (version 4.3.2). The network model was estimated using the IsingFit function from the IsingFit package, which employs the eLasso method combining l1-regularized logistic regression with model selection based on the Extended Bayesian Information Criterion (EBIC) and a default gamma parameter of 0.25 to identify relevant pairwise associations while penalizing model complexity. Network models were constructed using the qgraph package to calculate correlation coefficients and centrality indices (strength), with partial correlations controlling for confounding effects to estimate node associations. Bridge expected influence (BEI) was assessed via the networktools package to identify connections between MHKQ and PDD communities; BEI values reflect the sum of a node’s connections to the opposite community, where positive values indicate activating influences (enhancing co-occurrence) and negative values indicate deactivating influences (reducing co-occurrence) (17). Network stability was evaluated using the correlation stability coefficient (CS), where values >0.25 indicate acceptable stability and >0.5 suggest good stability. To identify potential intervention targets, the nodeIdentifyR package was used to perform simulation-based perturbations on the Ising model (18). This algorithm simulates an intervention by systematically altering the activation threshold of each node (by ±2 standard deviations) to make a specific symptom or belief harder or easier to trigger. Specifically, “aggravating” simulations mimic scenarios where misconceptions are entrenched or stress is increased, while “alleviating” simulations represent successful educational interventions that enhance knowledge accuracy. We then generated simulated samples based on these altered models and computed the resulting network sum scores. By plotting the mean sum scores with 95% confidence intervals, we ranked nodes based on their impact on the global network state, identifying which nodes, when modified, were associated with the most significant reduction (alleviating) or increase (aggravating) in overall stigma and knowledge deficits. Gender differences were compared using the NetworkComparisonTest package, testing invariance in global strength, expected influence, and edge weights (19).

## 3 Results

### 3.1 Participants and descriptive statistics

Descriptive statistics for MHKQ and PDD items are presented in **Table 2**. For MHKQ items (dichotomized as correct/incorrect responses), correct endorsement rates ranged from 13.61% (MHKQ13: “Adolescents’ psychological problems do not affect their academic performance”) to 92.10% (MHKQ1: “Psychological health is a component of overall health”). For PDD items (dichotomized as endorsement/non-endorsement of stigma, with “strongly agree/agree” coded as endorsement), endorsement ranged from 33.12% (Stigma5: “Most employers will not hire a person who has been hospitalized for mental illness”) to 65.23% (Stigma6: “Most people think less of a person after he/she has been hospitalized for a mental illness”). Centrality metrics, including expected influence (EI) and bridge expected influence (BEI), are also listed in **Table 2**, with EI values ranging from 3.326 (Stigma2) to 8.875 (MHKQ11) and BEI values ranging from -0.057 (MHKQ11) to 0.957 (MHKQ10).

**Table 2:** Descriptive statistics for MHKQ and stigma (PDD).

Characteristic			N = 12,537^1^	Centrality	
Node label	Item wording	Response		EI^2^	Bridge EI^3^
MHKQ1	Psychological health is a component of overall health.	Yes	11,547 (92.10%)	6.197	0.054
		No	990 (7.90%)		
MHKQ2	Mental illness is simply a problem of thought/ideology.	Yes	4,962 (39.58%)	4.79	0
		No	7,575 (60.42%)		
MHKQ3	Many people may have mental health problems but may not be aware of them.	Yes	9,683 (77.24%)	5.805	0.127
		No	2,854 (22.76%)		
MHKQ4	Mental illnesses are solely caused by psychological trauma or stimulation.	Yes	4,951 (39.49%)	5.271	0.039
		No	7,586 (60.51%)		
MHKQ5	Mental health includes normal intelligence, stable emotions, a happy mood, harmonious interpersonal relationships, and good adaptability.	Yes	9,876 (78.77%)	6.156	0.038
		No	2,661 (21.23%)		
MHKQ6	Most mental illnesses are incurable.	Yes	2,449 (19.53%)	5.689	0.748
		No	10,088 (80.47%)		
MHKQ7	If one suspects a mental health problem or mental illness, they should seek help from a psychotherapist or psychiatrist.	Yes	9,113 (72.69%)	6.059	0.18
		No	3,424 (27.31%)		
MHKQ8	People of almost any age can experience mental health problems.	Yes	9,498 (75.76%)	6.198	0.166
		No	3,039 (24.24%)		
MHKQ9	Mental illnesses and psychological problems cannot be prevented.	Yes	3,831 (30.56%)	5.037	0.122
		No	8,706 (69.44%)		
MHKQ10	Even for severe mental illnesses like schizophrenia, taking medication for a short period is sufficient; long-term continuous medication is unnecessary.	Yes	2,304 (18.38%)	7.5	0.957
		No	10,233 (81.62%)		
MHKQ11	An optimistic attitude, good interpersonal relationships, and healthy lifestyle habits help maintain mental health.	Yes	10,467 (83.49%)	8.875	-0.057
		No	2,070 (16.51%)		
MHKQ12	People with a family history of mental illness are more likely to develop mental health problems and disorders than the general population.	Yes	6,418 (51.19%)	6.648	0.181
		No	6,119 (48.81%)		
MHKQ13	Adolescents’ psychological problems do not affect their academic performance.	Yes	1,706 (13.61%)	6.161	0.53
		No	10,831 (86.39%)		
MHKQ14	The likelihood of middle-aged and elderly people developing psychological problems and mental disorders is very low.	Yes	2,195 (17.51%)	6.377	0.246
		No	10,342 (82.49%)		
MHKQ15	People with poor personality traits are more prone to developing psychological problems.	Yes	5,012 (39.98%)	5.684	0.182
		No	7,525 (60.02%)		
MHKQ16	High psychological stress or major life events (such as the death of a loved one) can easily trigger psychological problems and mental disorders.	Yes	8,374 (66.79%)	6.88	0.31
		No	4,163 (33.21%)		
MHKQ17	Do you know/Have you heard of World Mental Health Day?	Yes	6,520 (52.01%)	5.945	0.175
		No	6,017 (47.99%)		
MHKQ18	Do you know/Have you heard of International Day Against Drug Abuse (World Drug Day)?	Yes	11,290 (90.05%)	6.653	0.377
		No	1,247 (9.95%)		
MHKQ19	Do you know/Have you heard of World Suicide Prevention Day?	Yes	5,172 (41.25%)	6.313	0.305
		No	7,365 (58.75%)		
MHKQ20	Do you know/Have you heard of World Sleep Day?	Yes	5,550 (44.27%)	5.935	0.217
		No	6,987 (55.73%)		
Stigma1	Most people would accept a person who has been in a mental hospital as a close friend.	Yes	6,195 (49.41%)	4.517	0.158
		No	6,342 (50.59%)		
Stigma2	Most people believe that someone who has been hospitalized for mental illness is dangerous.	Yes	7,983 (63.68%)	3.326	0.565
		No	4,554 (36.32%)		
Stigma3	Most people believe that a person who has been hospitalized for mental illness is just as trustworthy as the average citizen.	Yes	6,500 (51.85%)	5.624	0.121
		No	6,037 (48.15%)		
Stigma4	Most people would accept a person who has fully recovered from mental illness as a teacher of young children in a public school.	Yes	6,454 (51.48%)	4.967	0.392
		No	6,083 (48.52%)		
Stigma5	Most employers will not hire a person who has been hospitalized for mental illness.	Yes	4,152 (33.12%)	6.206	0.609
		No	8,385 (66.88%)		
Stigma6	Most people think less of a person after he/she has been hospitalized for a mental illness.	Yes	8,178 (65.23%)	6.195	0.694
		No	4,359 (34.77%)		
Stigma7	Most people would be willing to marry someone who has been a patient in a mental hospital.	Yes	4,978 (39.71%)	5.427	0.07
		No	7,559 (60.29%)		
Stigma8	Most employers will hire a person who has been hospitalized for mental illness if he or she is qualified for the job.	Yes	5,559 (44.34%)	6.119	0.741
		No	6,978 (55.66%)		
Stigma9	Most people believe that entering a psychiatric hospital is a sign of personal failure.	Yes	7,479 (59.66%)	5.154	0.314
		No	5,058 (40.34%)		
Stigma10	Most people will not hire a person who has been hospitalized for serious mental illness to take care of their children, even if he or she had been well for some time.	Yes	7,986 (63.70%)	5.269	0.904
		No	4,551 (36.30%)		
Stigma11	Most people in my community would treat a person who has been hospitalized for mental illness just as they would treat anyone.	Yes	6,506 (51.89%)	4.708	0.224
		No	6,031 (48.11%)		
Stigma12	Most young people would be reluctant to date someone who has been hospitalized for a serious mental illness.	Yes	6,009 (47.93%)	5.695	0.106
		No	6,528 (52.07%)		

^1^n (%); ^2^Expected Influence; ^3^Bridge Expected Influence.

### 3.2 Network structure

**Figure 1:**
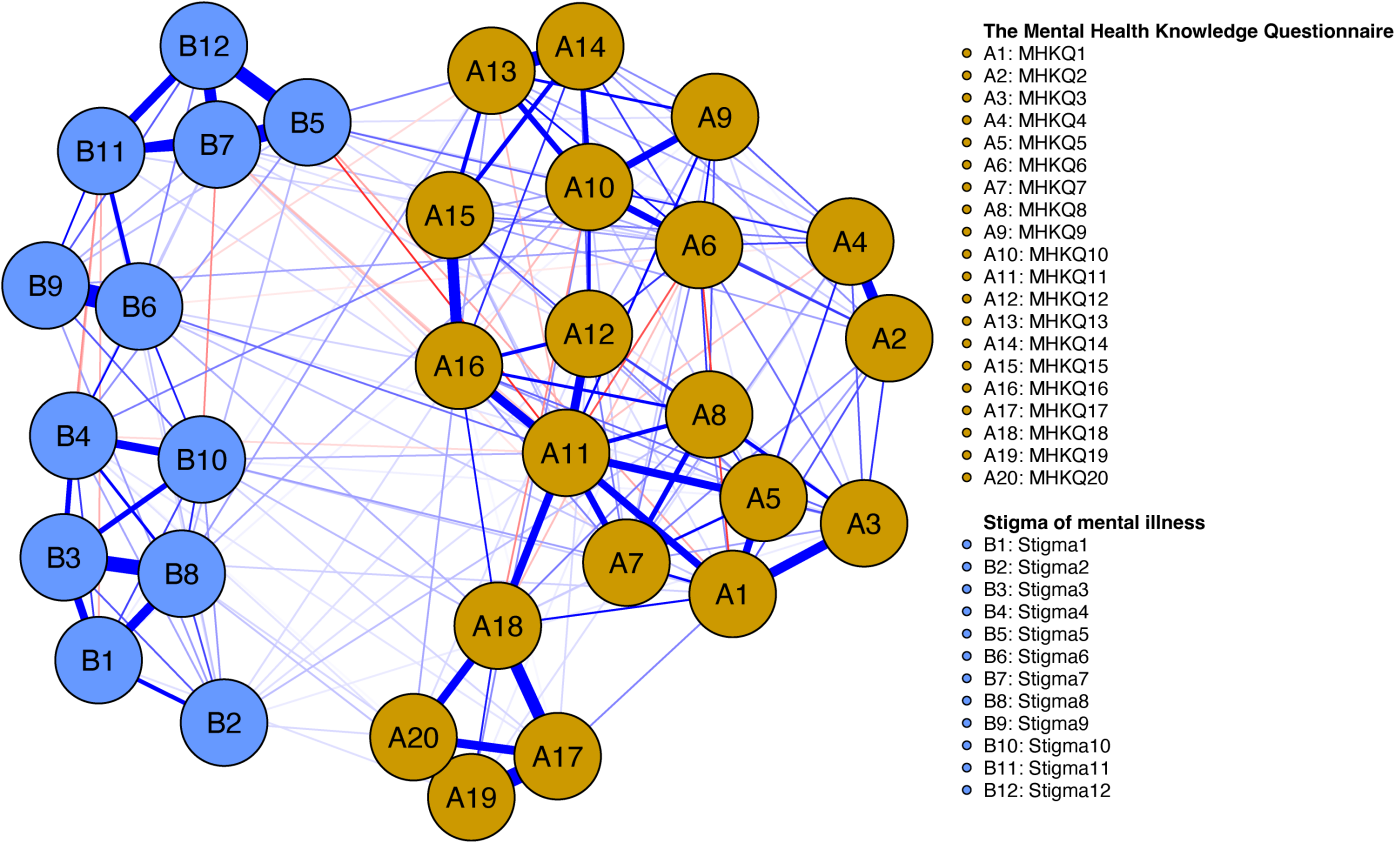
Association network of MHKQ and stigma (PDD).

**Figure 2:**
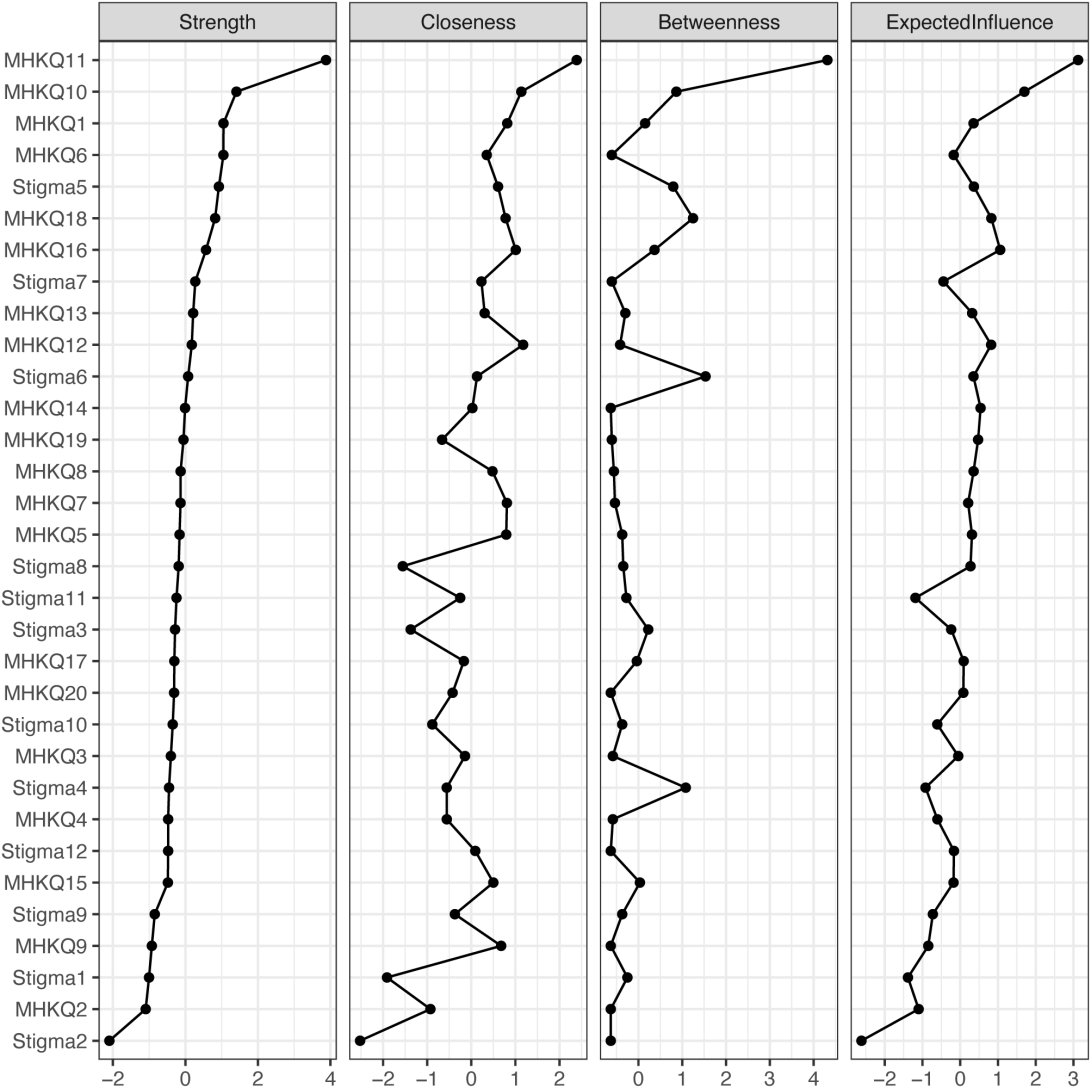
Bridge centrality indices of 32 nodes shown as standardized values z scores.

The network model, estimated using the IsingFit algorithm, depicted the associations among the 20 MHKQ items and 12 Stigma items in a circular layout (**Figure 1**), with MHKQ nodes forming one community and Stigma nodes another; edge thickness represented the strength of partial associations, while edge color indicated valence (blue for positive, red for negative). Dense intra-community connections were observed within the MHKQ cluster, particularly involving MHKQ11 (optimistic attitude, good relationships, and healthy habits help maintain mental health; threshold = -3.45), MHKQ10 (even for severe mental illnesses like schizophrenia, short-term medication suffices without long-term adherence; threshold = -4.25), and MHKQ6 (most mental illnesses are incurable; threshold = -3.60), while inter-community bridges were prominent, with MHKQ10 linking to Stigma5 (most employers will not hire a person who has been hospitalized for mental illness; threshold = -3.57), Stigma7 (most people would be willing to marry someone who has been a patient in a mental hospital), and Stigma12 (most young people would be reluctant to date someone who has been hospitalized for a serious mental illness; threshold = -2.38). Centrality indices identified influential nodes (**Figure 2**), with strength (indicating the sum of absolute edge weights connected to a node, reflecting direct connectivity) highest for MHKQ11 (12.06), MHKQ10 (8.45), and Stigma5 (7.74). Bridge expected influence (reflecting a node’s specific connectivity to the opposing community) emphasized MHKQ11, MHKQ10, and Stigma5.

### 3.3 Network stability

**Figure 3:**
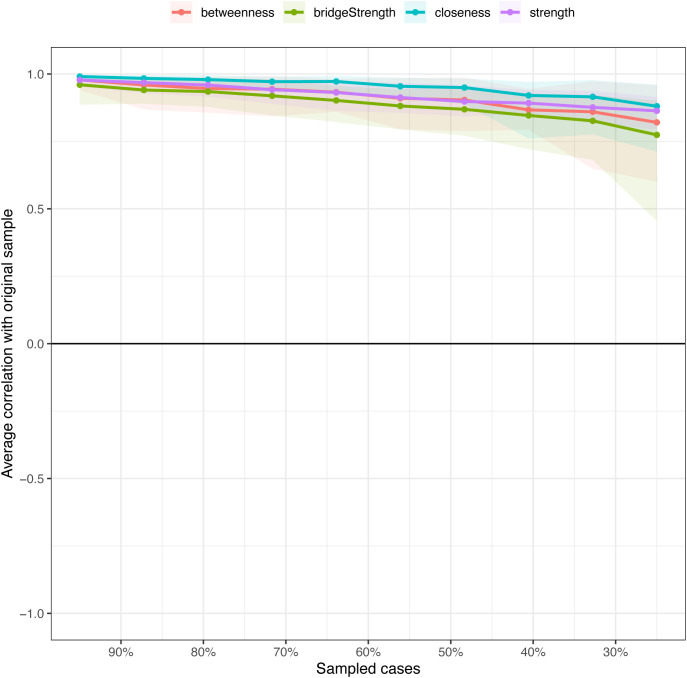
Stability of centrality indices by case dropping subset bootstrap.

The stability of the network was assessed using bootstrap methods with 500 replications and case-dropping bootstrap. The correlation stability (CS) coefficients indicated high stability for strength (CS > 0.75), edge weights (CS > 0.75), and intercepts (CS > 0.75). For bridge strength, the CS coefficients were 0.672, reflecting moderate stability. At a 75% sample drop (n=57), the network maintained correlations above 0.7 in 95% of the samples for strength, edge weights, and closeness (**Figures 3**).

### 3.4 Simulation of alleviating and aggravating interventions

**Figure 4:**
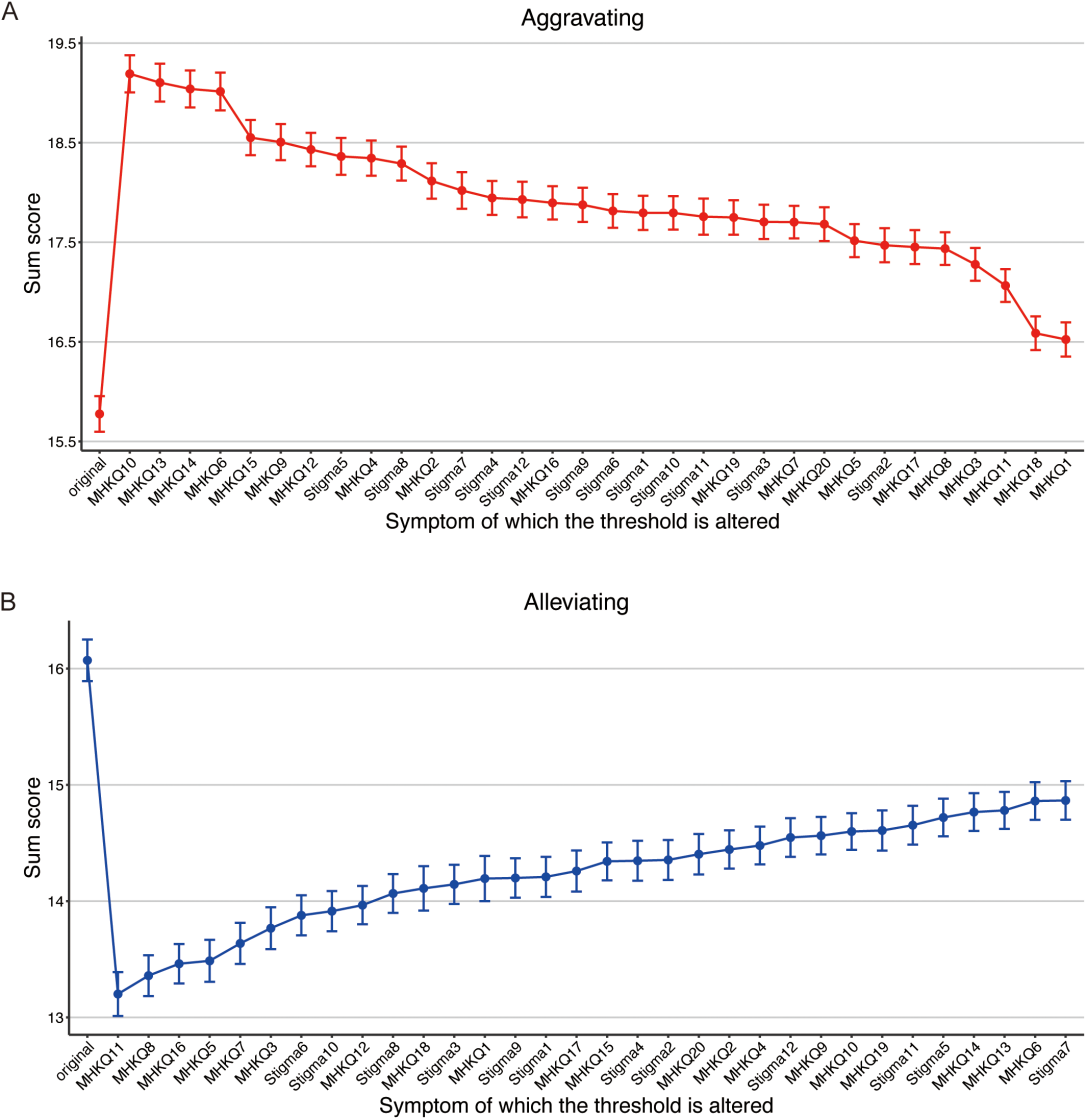
Simulation of aggravating **(A)** and alleviating **(B)**.

Threshold perturbations by ±2 standard deviations were simulated to evaluate node impacts on network sum scores (**Figures 4A, B**). For aggravating interventions (increasing thresholds), the highest mean sum scores were observed for MHKQ10 (19.36, 95% CI [19.18, 19.55]), MHKQ13 (19.09, [18.90, 19.28]; adolescents’ psychological problems do not affect their academic performance), and MHKQ14 (18.95, [18.77, 19.13]; middle-aged and elderly people have low likelihood of psychological problems and mental disorders). For alleviating interventions (decreasing thresholds), the lowest mean sum scores were recorded for MHKQ11 (13.20, [13.01, 13.39]), MHKQ8 (13.36, [13.18, 13.53]; almost anyone at any age can have psychological problems), and MHKQ16 (13.46, [13.29, 13.63]; high psychological stress or major events like death of a loved one easily trigger psychological problems and mental disorders).

### 3.5 Network comparisons

Network comparison tests between genders revealed non-significant differences in global strength invariance (16.28 for males vs. 15.91 for females; difference = 0.37, p = 0.693). Node strengths showed minimal variations, with non-significant p-values for MHKQ11 (p = 0.588; optimistic attitude, good relationships, and healthy habits help maintain mental health), MHKQ10 (p = 0.864; even if diagnosed with severe mental illness like schizophrenia, taking medication for a period is enough, no need for long-term uninterrupted medication), and Stigma5 (p = 0.946; most employers will not hire a person who has been hospitalized for mental illness). Expected influences were similarly invariant, with p-values exceeding 0.097 for MHKQ3 (p = 0.097; many may have unnoticed mental health issues), MHKQ5 (p = 0.097; mental health includes normal intelligence, stable emotions, harmonious relationships, and good adaptability), and MHKQ20 (p = 0.186; awareness of World Sleep Day). Edge invariance tests confirmed structural similarity, with non-significant p-values for key connections such as MHKQ11–MHKQ10 (p = 0.898), MHKQ10–Stigma5 (p = 0.272), and MHKQ11–Stigma7 (p = 0.330).

## 4 Discussion

The current study represents one of the first applications of network analysis to elucidate the intricate interconnections between mental health knowledge (MHK) and perceived stigma among Chinese high school students. By modeling MHK and stigma as dynamic networks, we identified key nodes and bridges that may serve as pivotal targets for intervention, while also examining gender invariance to inform broadly applicable strategies. Our findings align with emerging evidence from network-based approaches in psychiatric epidemiology, highlighting how misconceptions in MHK are closely linked to stigmatizing perceptions, thereby potentially perpetuating barriers to help-seeking in this demographic (3, 6–8).

Central to our network was MHKQ11 (“optimistic attitude, good relationships, and healthy habits help maintain mental health”), which exhibited the highest strength, underscoring its role as a hub within the MHK community. This node’s prominence suggests that promoting positive lifestyle factors might have widespread associations within the network, enhancing overall MHK and potentially relating to lower stigma. Similarly, MHKQ10 (“even for severe mental illnesses like schizophrenia, short-term medication suffices without long-term adherence”) emerged as a critical bridge to stigma items, such as Stigma5 (perceived hiring discrimination) and Stigma7 (reluctance to marry former patients). These connections reflect how erroneous beliefs about treatment chronicity are associated with heightened perceptions of social exclusion and discrimination, consistent with prior studies on mental health literacy in Chinese adolescents, where low awareness of evidence-based treatments correlates with heightened public stigma (2, 9). The dense intra-community ties within MHK, particularly involving misconceptions about incurability (MHKQ6) and vulnerability across ages (MHKQ8, MHKQ14), further illustrate how knowledge deficits cluster and are interconnected, mirroring network patterns observed in symptom networks of depression among youth (10).

Simulation-based perturbations provided actionable insights into intervention priorities, revealing how threshold adjustments (± 2 standard deviations) on individual nodes are associated with changes in the overall network sum scores, which represent aggregate activation levels of MHK deficits and stigma. In aggravating interventions (increasing thresholds to simulate entrenched misconceptions), the most pronounced escalations occurred for MHKQ10 (mean sum score: 19.36, 95% CI [19.18, 19.55]), MHKQ13 (“adolescents’ psychological problems do not affect their academic performance”; 19.09, [18.90, 19.28]), and MHKQ14 (“middle-aged and elderly people have low likelihood of psychological problems”; 18.95, [18.77, 19.13]). These nodes, particularly MHKQ10, likely contribute to higher network activation because they connect core treatment myths to practical implications in high-stakes contexts like education and aging, potentially worsening stigma by reinforcing perceptions of mental illness as irreversible or irrelevant to daily functioning. This pattern suggests that unaddressed errors in these areas could be linked to widespread activation, akin to how central symptoms in depression networks underpin comorbidity in stressed populations (20). Conversely, alleviating interventions (decreasing thresholds to mimic knowledge enhancement) produced the greatest reductions via MHKQ11 (13.20, [13.01, 13.39]), MHKQ8 (“almost anyone at any age can have psychological problems”; 13.36, [13.18, 13.53]), and MHKQ16 (“high psychological stress or major events easily trigger psychological problems”; 13.46, [13.29, 13.63]). Targeting MHKQ11, for example, may efficiently deactivate the network by leveraging its high centrality to promote a cascade of positive corrections, normalizing mental health maintenance and potentially lowering linked stigma. These differential effects underscore the value of node-specific simulations in identifying leverage points: aggravating highlights vulnerabilities to misinformation, while alleviating prioritizes protective factors, extending prior applications in stigma and psychopathology networks where such dynamics inform tailored, cost-effective interventions (21). In the Chinese context, where academic performance is paramount, addressing MHKQ13’s link to stigma may be particularly salient, as it counters the cultural minimization of mental health’s impact on scholastic outcomes, potentially acting against internalized stigma among students facing gaokao pressures.

Notably, the differential impacts observed in these simulations (where MHKQ10 showed the strongest aggravating effect while MHKQ11 led in alleviating outcomes) highlight a key distinction between static network topology and dynamic intervention effects. While MHKQ11 demonstrated the strongest centrality metrics, indicating its extensive direct connections (high strength), the threshold perturbations simulate real-world changes in node activation thresholds, modeling how altering specific beliefs might theoretically cascade through the system (20, 21). For instance, MHKQ11’s central position arises from its broad ties to multiple MHK items, reflecting a foundational belief in preventive behaviors that underpins broader knowledge structures. However, in interventions, MHKQ10 showed the strongest aggravating association in the simulation, likely due to its bridging function between MHK misconceptions and stigma perceptions; reinforcing this node (e.g., perpetuating myths about treatment non-adherence) may be linked to disproportionate activation of stigma clusters by linking curability doubts directly to devaluation attitudes. In contrast, MHKQ11’s alleviating potential stems from its hub status, allowing corrections in positive habit awareness to broadly deactivate interconnected deficits. Furthermore, integrating the estimated thresholds from the Ising model provides additional insight: MHKQ11 has a threshold of -3.45, less negative than MHKQ10’s -4.25, suggesting higher baseline correct response rates for MHKQ11 (assuming coding where 1=correct knowledge endorsement, with negative thresholds biasing toward 0=incorrect, but less so for MHKQ11). This implies MHKQ11 already enjoys higher accuracy among participants, leaving less room for improvement through intervention compared to MHKQ10, where lower correct rates (more negative threshold) offer greater potential for impactful change, aligning with principles in network interventions where nodes with suboptimal baseline activation yield larger system-wide shifts upon targeting (20).

Moreover, our findings lend empirical support to the Enhanced Contact Model (ECM) proposed by Ran et al. (22), which posits that structured, positive contact (augmented by education and knowledge dissemination) can help reduce stigma associated with mental illness. The identified bridges between MHK nodes (e.g., MHKQ10 and MHKQ11) and stigma perceptions demonstrate how targeted knowledge enhancement may help mitigate stigmatizing attitudes, aligning with ECM’s emphasis on combining interpersonal contact with informational strategies to foster attitude change and decrease devaluation. This network-level evidence suggests that interventions grounded in ECM, such as peer-led education sessions in schools, could enhance the potential impact of alleviating key knowledge deficits, thereby promoting sustained reductions in perceived stigma among adolescents.

Gender comparisons revealed invariant network structures, with non-significant differences in global strength, node centrality, and edge weights, implying that MHK-stigma dynamics operate similarly across males and females. This null finding regarding structural differences has important practical implications: it suggests that distinct intervention protocols for male and female students may not be necessary. Instead, a universal, standardized mental health curriculum targeting the identified central nodes (e.g., MHKQ11) and bridges (e.g., MHKQ10) is likely to be equally effective across genders, simplifying the implementation of large-scale school-based programs. This invariance contrasts with some gender-specific findings in stigma internalization (9), but supports the universality of core misconceptions in Chinese youth, possibly due to shared educational and cultural exposures. Nonetheless, subtle variations in expected influence (e.g., MHKQ3 and MHKQ5) warrant further exploration in longitudinal designs to capture potential developmental divergences (19, 20).

Our network’s high stability (CS coefficients >0.75) bolsters confidence in these findings. The use of Ising models and partial correlations advances beyond traditional latent variable approaches, revealing nuanced pathways overlooked in aggregate scoring of MHKQ and PDD (2). However, limitations must be acknowledged. First, the cross-sectional design precludes causal inferences; future studies should employ longitudinal networks to track changes post-intervention. Second, as the MHKQ and PDD are self-report measures, responses may be subject to social desirability bias, where students might underreport stigmatizing attitudes or overreport knowledge to align with perceived social norms. Third, the study was conducted in Sichuan Province, which may limit the generalizability of findings to other regions in China with different socioeconomic characteristics.

These findings have profound implications for public health in China, aligning with national initiatives like the Healthy China 2030 blueprint to enhance adolescent mental health literacy. School-based interventions could prioritize MHKQ11 and MHKQ10 through psychoeducation modules, potentially integrated with digital platforms to reach high-pressure environments. By targeting bridges, such programs may help interrupt stigma maintenance, fostering help-seeking and reducing associated outcomes like non-suicidal self-injury (1). Future research should extend network analyses to include environmental factors and compare with international cohorts to delineate culture-specific patterns.

## Data Availability

The raw data supporting the conclusions of this article will be made available by the authors, without undue reservation.
