# Supplementary material for: Network analysis of mental health knowledge and stigma among high school students in Sichuan, China
Source: Front Psychiatry. 2026 Jan 29;17:1728556. doi: 10.3389/fpsyt.2026.1728556 (PMC12894237; doi:10.3389/fpsyt.2026.1728556)
Supplement: Supplementary file 1 [file Supplementaryfile1.docx]

Supplementary Material**
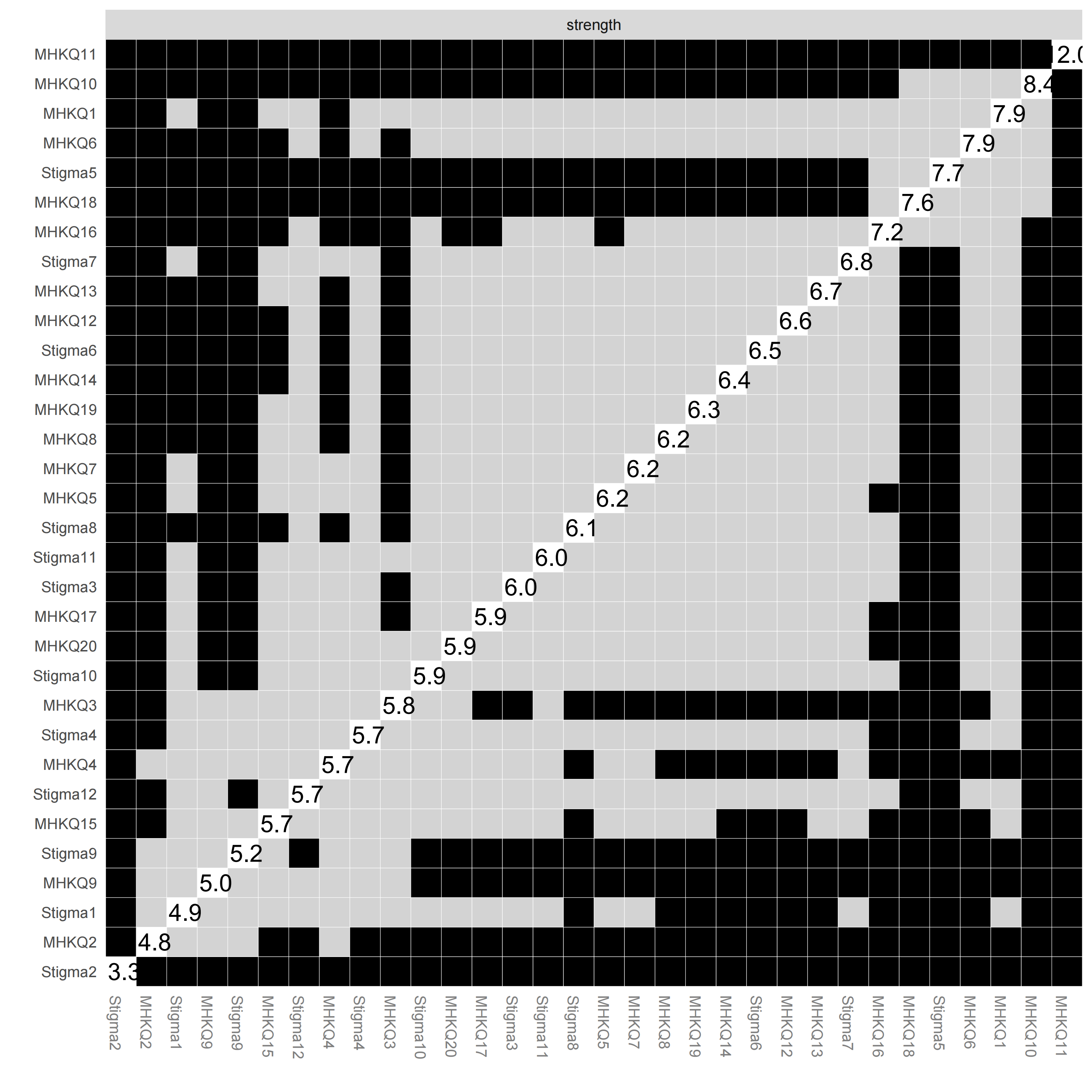
**

Supplementary Figure 1: Bootstrap Stability of Strength Centrality. This plot shows the bootstrapped confidence intervals for node strength centrality, ordered by sample values.


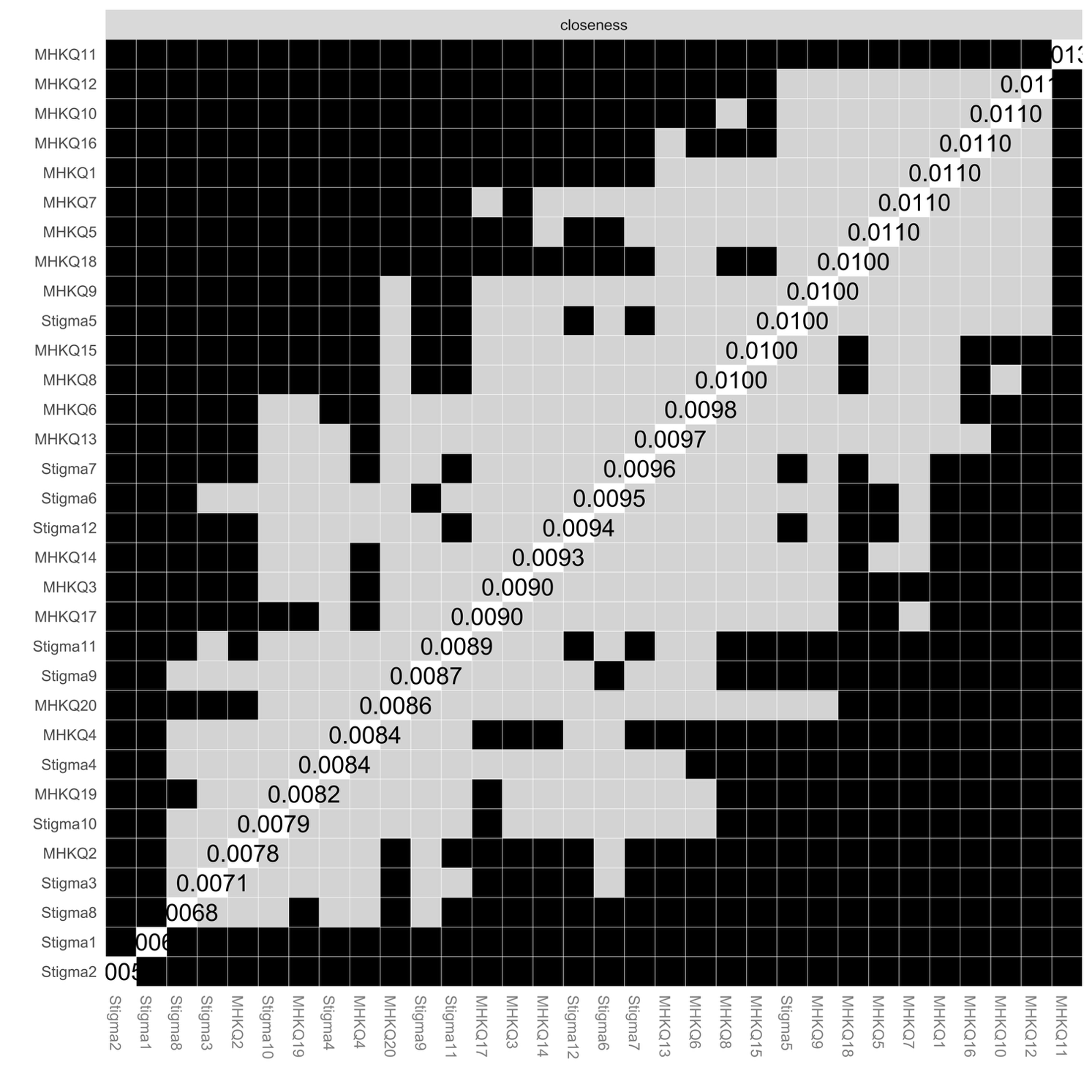


**Supplementary Figure 2:** Bootstrap Stability of Closeness Centrality. This plot displays the bootstrapped confidence intervals for node closeness centrality.


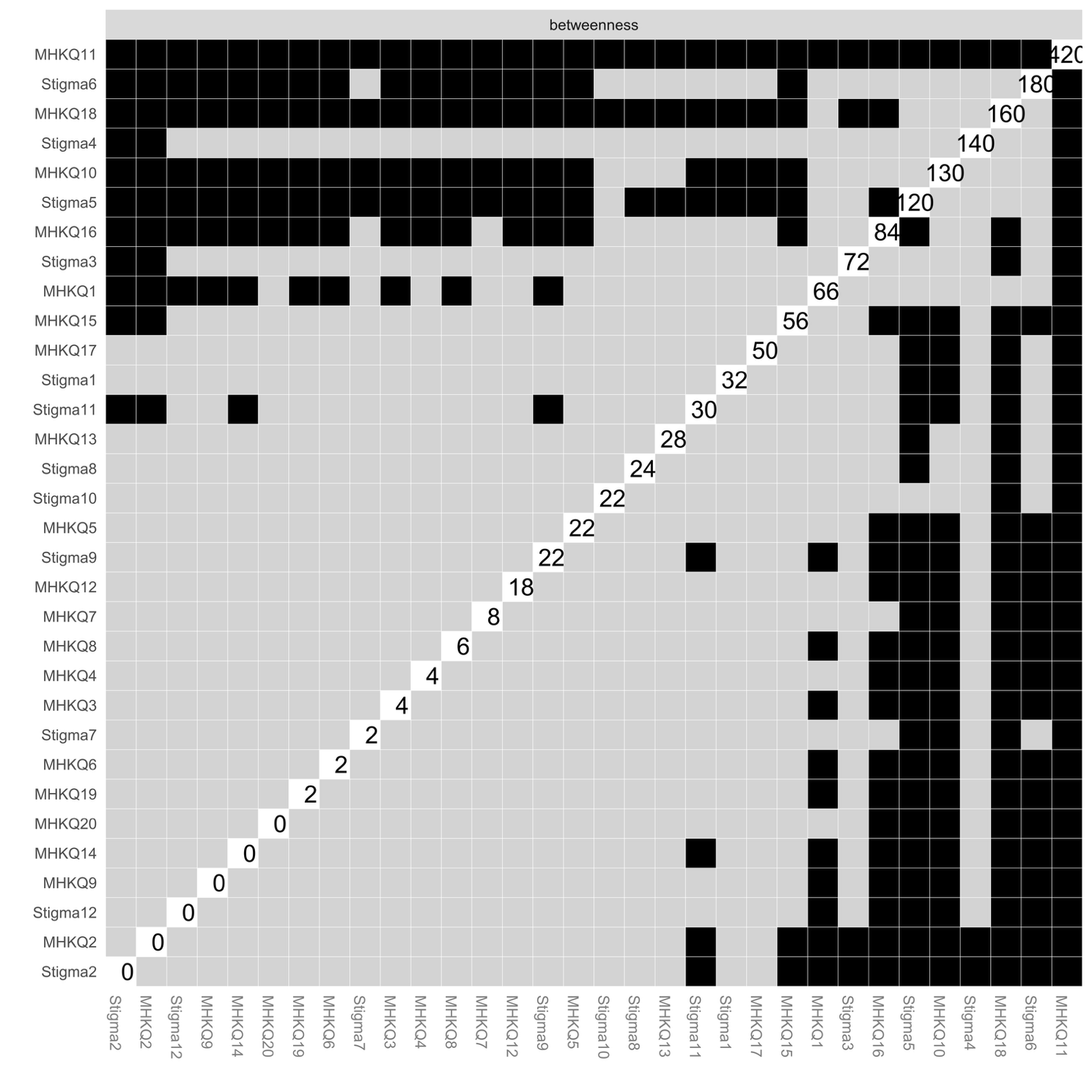


**Supplementary Figure 3:** Bootstrap Stability of Betweenness Centrality. This plot illustrates the bootstrapped confidence intervals for node betweenness centrality.


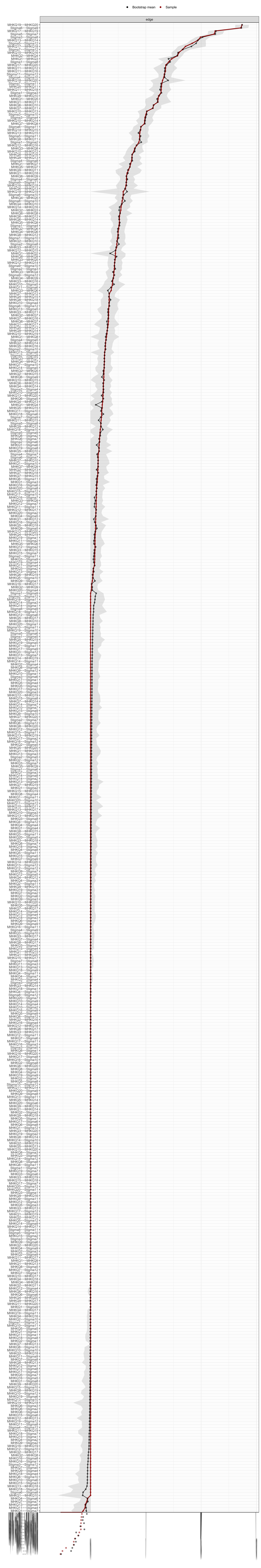


**Supplementary Figure 4:** Bootstrap Stability of Edge Weights. This plot presents the bootstrapped confidence intervals for edge weights in the network.


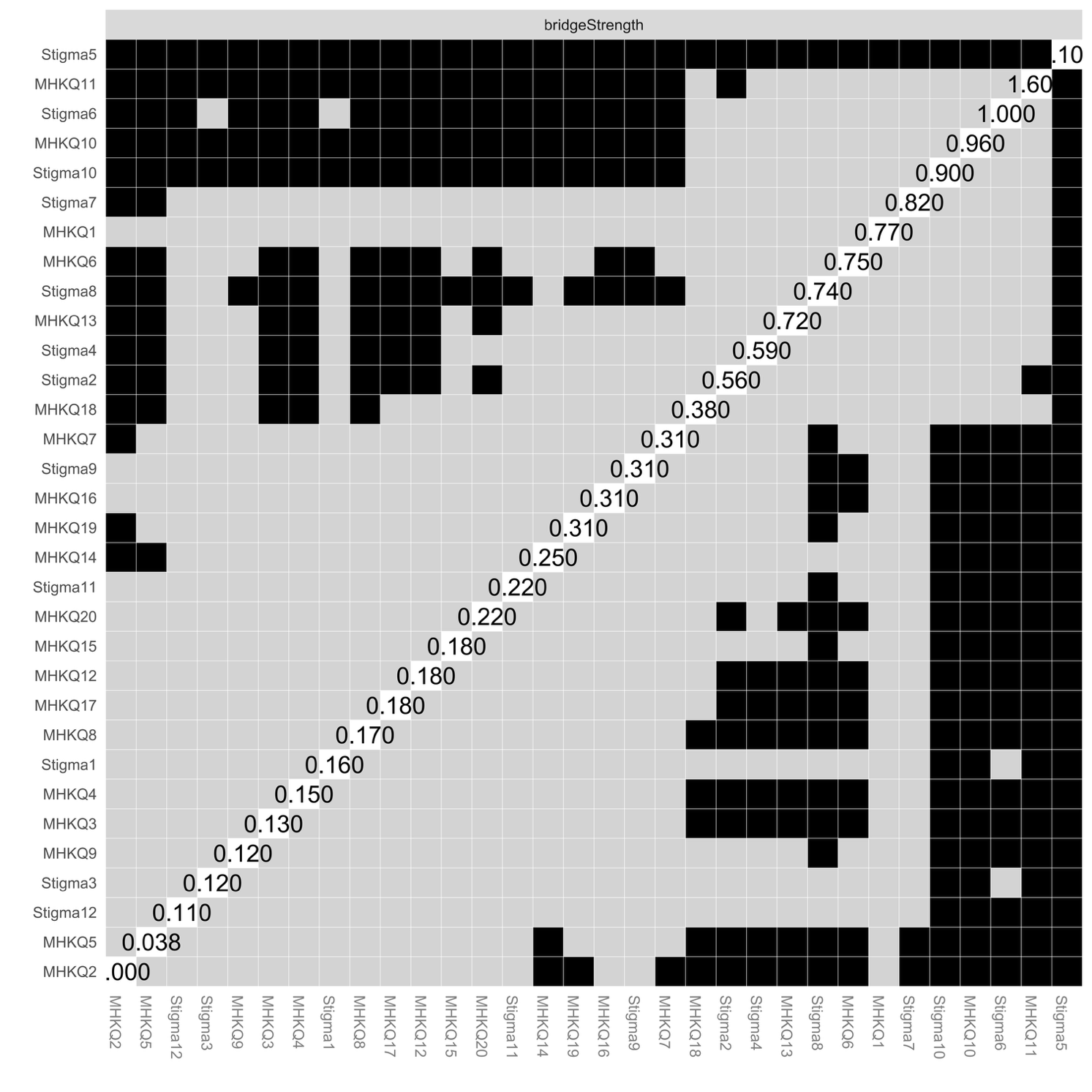


**Supplementary Figure 5:** Bootstrap Stability of Bridge Strength. This plot shows the bootstrapped confidence intervals for bridge strength, highlighting the stability of inter-community connections.
